# Impact of sleep quality on work-related musculoskeletal disorders among nurses: a structural equation model integrating psychosocial factors

**DOI:** 10.3389/fpubh.2026.1881980

**Published:** 2026-07-15

**Authors:** Lai Lichong, Dong Pengxin, Pan Qini, Peng Jie, Wu Haichen, Li Zhixin, Huang Caiyan, Chai Yidan, Huang Ping, Liu Haowei, Zeng Jingyun, Zhou Huimin, Huang Huiqiao, Zhou Dongna, Liao Yifen

**Affiliations:** 1 The Second Affiliated Hospital of Guangxi Medical University, Nanning, Guangxi, China; 2 The First People's Hospital of Yulin, Yulin, Guangxi, China; 3 Nanxishan Hospital of Guangxi Zhuang Autonomous Region, Guilin, Guangxi, China

**Keywords:** nurse, sense of agency, sleep quality, social support, work-related musculoskeletal disorders

## Abstract

**Background and objective:**

Work-related musculoskeletal disorders (WMSDs) are highly prevalent among nurses and threaten workforce stability. Although sleep disturbance is known to be associated with WMSDs, the psychosocial pathways underlying this relationship remain poorly understood. This study aims to examine whether sense of agency and perceived social support mediate the relationship between sleep quality and WMSDs among hospital nurses, using a structural equation modeling approach.

**Methods:**

A convenience sample of staff nurses was recruited from six tertiary hospitals in three Chinese cities. Participants self-reported sociodemographics, the Pittsburgh Sleep Quality Index (PSQI), sense of personal control, perceived social support, and the 12-month prevalence/number of WMSDs. Structural equation modeling with maximum likelihood estimation was constructed to examine the hypothesized pathways, and bias-corrected bootstrap analysis was conducted to analyze the mediating effect of the sense of agency and perceived social support between sleep quality and the number of WMSDs.

**Results:**

Our study included information from 1,388 nurses working in clinical settings. After controlling the factors of gender, length of service, night shift, frequent trunk flexion, frequent heavy lifting, and perceived work fatigue, the PSQI of nurses showed a direct negative association with the sense of agency and perceived social support. Perceived social support had a direct positive impact on the sense of agency, and perceived social support and agency had a direct negative impact on the number of WMSDs in nurses (*p* < 0.001), χ2/df = 7.466, CFI = 0.906, RMSEA = 0.070, and the model demonstrated acceptable fit indices. The direct effect accounted for 81.8% of the total effect, while the indirect effects through three significant mediation paths accounted for 18.2% of the total effect (indirect effect = 0.035, total effect = 0.192).

**Conclusion:**

Even after accounting for established ergonomic and occupational covariates, poor sleep quality was not only directly associated with a higher number of WMSD sites among nurses but also showed an indirect association with WMSDs through lower sense of agency and perceived social support. Interventions that protect and bolster these psychosocial resources should be embedded within integrated musculoskeletal-health frameworks addressing biological, psychological, and social determinants.

## 1 Background

Global population aging and the concomitant surge in healthcare demand have driven a continuous expansion of the nursing workforce (1). By the end of 2025, the total number of registered nurses in China will reach 6.062 million, an increase of about 1.35 million or 29% compared to 2020. However, this quantitative expansion has not resolved the persistent challenges of workforce shortages and high turnover rates that continue to plague the nursing profession globally. Nurses experience elevated rates of occupational injuries, burnout, and job dissatisfaction, which not only compromise patient care quality but also drive experienced nurses away from clinical practice (2). Therefore, addressing nurses’ health and occupational protection needs is integral to retaining skilled personnel and ensuring the sustainable development of healthcare systems. Work-related musculoskeletal disorders (WMSDs) are defined as discomfort, pain, numbness or other symptoms lasting ≥24 h in one or more major body regions that are attributable to work or the work environment and that do not resolve after rest. The annual prevalence of WMSDs among nurses is as high as 79% (3), significantly exceeding that of other healthcare professionals and the general working population (4, 5). These disorders not only impair nurses’ work performance and increase absenteeism, but may also precipitate chronic pain and burnout, creating a vicious cycle of health deterioration and declining service capacity (6). Consequently, elucidating the mechanisms underlying the onset and progression of WMSDs and constructing a scientifically sound intervention system are of paramount importance.

Shift-work schedules inherent to nursing inevitably disrupt circadian rhythms and precipitate chronic sleep loss. Epidemiological data indicate that the prevalence of shift-work sleep disorder among nurses reaches 45.5% (7). Skeletal muscle harbors the largest peripheral clock network in the human body, and both central and peripheral clocks orchestrate the crosstalk between the musculoskeletal system and energy metabolism. Deteriorated sleep quality accelerates fatigue accumulation, attenuates attention and blunts recovery capacity, thereby predisposing nurses to musculoskeletal imbalance and injury under high workloads (8). In parallel, sleep restriction directly interferes with pain-modulatory circuits, lowers pain threshold and amplifies the subjective experience of musculoskeletal discomfort (9). Consequently, we positioned the Pittsburgh Sleep Quality Index at the entry point of our causal chain to examine how circadian misalignment induced by shift work influences WMSDs. We advance the following hypothesis:

*H1:* Nurse sleep quality index has a positive effect on WMSDs.

Earlier WMSD studies focused predominantly on biomechanical risks and physical-environment factors, whereas psychosocial variables such as job stress, emotional exhaustion and perceived organizational support have recently gained visibility in occupational-health research. Sleep quality directly shapes nurses’ somatic and mental states, thereby initiating a chain reaction that influences their psychological and behavioral performance at work (10). Concurrent evidence shows that psychosocial job characteristics—namely support, cooperation, job control and psychological demands—are significantly associated with musculoskeletal morbidity (11). We therefore adopt psychosocial factors as our entry point and undertake a systematic, empirical examination of their interplay with physiological indices such as sleep quality in a nursing cohort.

Sense of Agency (SoA) refers to the subjective experience, generated during activity, that one can control one’s own behavior and, through that behavior, influence the course of external events; it represents individual competence and is closely linked to well-being, health status and anxiety, rendering it a valid predictor of mental-health outcomes (12). Adequate sleep is the physiological foundation that sustains cognitive function, emotional stability and self-regulatory capacity (13, 14). Research indicates that poor sleep quality undermines executive control and decision-making ability, thereby diminishing nurses’ perceived autonomy and mastery at work (15). Moreover, perceived competence is a key determinant of nurses’ health and performance; nurses with low SoA are more likely to lapse into passive coping, develop burnout and exhibit blunted risk perception, all of which elevate the incidence of repetitive-strain injuries and chronic pain (16). On the basis of these convergent findings we advance the following hypotheses:

*H2:* The sleep quality index of nurses has a negative effect on the sense of agency.

*H3:* The sense of agency among nurses has a negative impact on WMSDs.

Second, poor sleep quality may also impair nurses’ perception of social support. Perceived social support denotes the care, assistance and backing that individuals subjectively experience from others in their social environment—colleagues, supervisors, family members and the like. Augmenting perceived support, especially through refined leadership and management strategies, buffers the impact of burnout on depressive symptoms and exerts a salutary influence on nurses’ health and performance (17). Nurses afflicted by sub-optimal sleep are often physically and mentally exhausted; this fatigue blunts their sensitivity to available support, so that when confronted with workplace difficulties they are more inclined to endure hardship alone rather than seek or utilize assistance. Such withdrawal not only amplifies psychological burden but also erodes work engagement (18). Robust social support can directly mitigate the physiological tension and muscular load imposed by job stress (19), whereas its absence fosters social isolation, reduces job satisfaction and increases the adoption of awkward working postures, thereby intensifying chronic strain on the musculoskeletal system (20). Accordingly, we propose the following hypotheses:

*H4:* Nurse sleep quality index has a negative impact on perceived social support.

*H5:* Nurses’ perceived social support has a negative impact on WMSDs.

Literature reviews consistently identify gender, length of employment, shift-work status, awkward working postures and perceived work fatigue as salient determinants of WMSDs among nurses (3). Incorporating these variables as controls enables us to partial out their confounding influence and to isolate the independent pathways linking sleep quality and psychosocial factors to WMSD morbidity. Profiling the differential impact of each covariate also furnishes a theoretical basis for targeted, tiered occupational-health interventions. Despite growing evidence linking sleep disturbance to WMSDs, several important gaps remain. First, most prior studies have examined psychosocial factors (e.g., social support, job control) and sleep quality as independent predictors, without integrating them into a unified mediation framework. Second, although sense of agency has been linked to psychological well-being, its role as a mediator between sleep quality and WMSDs has not been empirically tested among nurses. Third, conventional regression approaches cannot simultaneously estimate direct and indirect pathways, limiting understanding of the relative magnitude of these effects. To address these gaps, this study constructs a theory-driven structural equation model (SEM) that integrates sleep quality, perceived social support, and sense of agency to predict WMSDs among nurses, and provides a scientific basis for ensuring the musculoskeletal health of nurses and the sustainable development of nurses.

## 2 Subjects and methods

### 2.1 Study participants

Convenience sampling was used to recruit clinical nurses from six tertiary hospitals in three cities of Guangxi, China. Within each hospital, department head nurses identified eligible nurses, who were then invited to complete either a paper questionnaire during shift breaks (offline) or an electronic questionnaire via the hospital’s internal communication platform (online). (1) Inclusion criteria: ① possession of a valid nursing license and ≥ 1 year of independent clinical practice; ② Currently engaged in direct patient care for ≥20 h per week; ③ understanding of the study aims and voluntary participation. (2) Exclusion criteria: ① nurses temporarily off-site (e.g., on external training, maternity or sick leave); ② pregnancy or breastfeeding within the past year; ③ congenital spinal disease, tumor, gynecological disorders or any other non-occupational conditions causing musculoskeletal pain; ④ definite history of trauma or surgery within the past year; ⑤ musculoskeletal complaints attributable to regular exercise, domestic overexertion. The study protocol was approved by the institutional ethics committee (Approval No. 2023-KY-0941).

### 2.2 Survey contents

#### 2.2.1 WMSD-related factor questionnaire

Evidence-based variables associated with WMSDs were collected: age, sex, marital status, BMI, weekly exercise frequency, length of service, department, shift-work status, frequent trunk flexion, frequent heavy lifting, and perceived job fatigue.

#### 2.2.2 Work-related musculoskeletal disorders

The Nordic Musculoskeletal Questionnaire (NMQ) was used. Nine anatomical regions (neck, shoulder, upper back, lower back, elbow, wrist/hand, hip/thigh, knee, ankle/foot) were assessed. Each region was scored 0 = no symptoms, 1 = symptoms present; occurrence in ≥1 region was defined as a case. Higher summed scores indicate a greater number of affected sites. Cronbach’s *α* for the scale in the present sample was 0.861.

#### 2.2.3 Pittsburgh Sleep Quality Index

The PSQI scale consists of seven dimensions: subjective sleep quality, sleep latency, sleep duration, sleep efficiency, sleep disturbance, sleep medication use, and daytime dysfunction. It is commonly used to measure participants ‘sleep quality over the previous month. Total scores range 0–21; > 5 denotes poor sleep quality. Cronbach’s *α* in this study was 0.784.

#### 2.2.4 Perceived Social Support Scale

The 12-item PSSS comprises three 4-item subscales: work support, family support and friend support. Items are rated on a 7-point Likert scale (1 = strongly disagree, 7 = strongly agree); higher scores indicate greater support. Cronbach’s α values for work, family, friend subscales and the total scale were 0.965, 0.952, 0.973 and 0.968, respectively.

#### 2.2.5 Sense of Agency Scale

The 9-item Sense of Agency Scale developed by Tapal (21) was used after translation and validation in Chinese. Items are rated 1–7; items 2, 3, 6 and 7 are reverse-scored. Total scores range 9–63, with higher values reflecting stronger sense of agency. Cronbach’s *α* in the present study was 0.832.

### 2.3 Statistical analysis

Descriptive statistics were generated with SPSS 23.0. Continuous variables with a normal distribution are presented as mean ± SD and were compared between groups using independent-samples *t* tests or one-way ANOVA; non-normally distributed continuous variables are expressed as median (inter-quartile range) and were compared with Mann–Whitney U or Kruskal–Wallis tests. Categorical data are described as frequencies and percentages, and group differences were examined with the χ2 test or Fisher’s exact test to identify factors associated with the number of WMSD sites among shift-working nurses. All tests were two-tailed; *p* < 0.05 was considered statistically significant.

On the basis of the literature, sleep quality, perceived social support, sense of agency and WMSD status were specified as path variables. Prior to modeling, Harman’s single-factor test was applied to assess common-method bias, and multicollinearity diagnostics were conducted. Pearson correlations in SPSS were used for preliminary exploration of variable relationships. The sleep–psychosocial–WMSD structural equation model for shift nurses was estimated with the lavaan package in R. Model fit was evaluated against *a priori* criteria commonly applied in structural equation modeling: CFI ≥ 0.90, RMSEA ≤0.08, and χ^2^/df < 5.0 as indicators of acceptable fit, with TLI ≥ 0.90 as a more stringent criterion.

## 3 Results

### 3.1 Prevalence of WMSDs and univariate analysis

A total of 1,388 clinical nurses were surveyed; mean age was 30.83 ± 6.73 years. The overall prevalence of WMSDs was 83.56%, and the mean number of affected anatomical sites was 3.87 ± 2.87. Univariate analysis revealed statistically significant differences in the number of WMSD sites across the following variables: age, gender, physical exercise, length of service, department, shift-work status, frequent trunk flexion, frequent heavy lifting and perceived work fatigue (Table 1).

**Table 1:** Single factor analysis of general data of nurses and prevalence of WMSDs.

Item	Basic information	Number of WMSD sites	t/*F*/Z	*P*
Age	30.83 ± 6.73	3.87 ± 2.87	0.102	<0.001
Gender			−2.370	0.008
Male	102 (7.62)	3.23 ± 2.51		
Female	1,236 (92.38)	3.93 ± 2.90		
Marital status			−0.026	0.983
Married	704 (52.62)	3.89 ± 2.84		
Single/divorced/widowed	634 (47.38)	3.85 ± 2.91		
BMI	20.9 ± 3.01	3.87 ± 2.87	−0.014	0.609
Weekly exercise frequency			3.570	0.028
0	656 (49.03)	4.04 ± 2.87		
1–3	564 (42.15)	3.63 ± 2.78		
>3	118 (8.02)	4.10 ± 3.23		
Length of service	9.15 ± 6.91	3.87 ± 2.87	0.093	<0.001
Department			10.000	<0.001
Internal medicine	407 (30.42)	4.45 ± 2.90		
Surgery	371 (27.73)	4.16 ± 2.74		
ICU	140 (10.46)	3.84 ± 2.59		
Emergency	90 (6.73)	2.74 ± 2.56		
Pediatrics	104 (7.77)	2.80 ± 3.14		
Obstetrics and gynecology	106 (7.92)	3.88 ± 3.02		
Operating room	75 (5.61)	3.01 ± 2.51		
Outpatient	45 (3.36)	2.44 ± 2.78		
Shift work			−3.123	0.002
Yes	1,080 (80.72)	3.99 ± 2.91		
No	258 (19.28)	3.37 ± 2.65		
Frequent trunk flexion			13.523	<0.001
Yes	1,037 (77.50)	4.41 ± 2.82		
No	301 (22.50)	2.02 ± 2.23		
Frequent heavy lifting			7.582	<0.001
Yes	681 (50.90)	4.44 ± 2.88		
No	657 (49.50)	3.28 ± 2.75		
Perceived work fatigue			159.706	<0.001
Not fatigued	183 (13.68)	1.91 ± 2.46		
Somewhat fatigued	465 (34.08)	2.75 ± 2.26		
Quite fatigued	580 (43.35)	4.78 ± 2.59		
Extremely fatigued	110 (8.22)	7.11 ± 2.64		

### 3.2 Common method bias test and multicollinearity test

In order to control the common method bias problem, our study uses the Harman single factor method to test. The results showed that the first factor without rotation explained 23.82% of the variation, and did not account for the critical value of 40.00% of the total variation. Therefore, it is considered that there is no obvious common method bias in our study. Multiple collinearity tests were performed on the included control variables (gender, working years, night shift, frequent trunk flexion, frequent heavy lifting, perceived work fatigue), PSQI, perceived social support, and sense of agency. The results showed that VIF was < 5, and multiple collinearity was acceptable.

### 3.3 Correlation analysis between variables

Pearson correlation analysis showed that PSQI, perceived social support, and sense of agency were all related to the number of WMSD affected sites (*p* < 0.001), as shown in Table 2.

**Table 2:** Correlation analysis between variables.

Item	Sleep quality index	Perceived social support	Sense of agency
Perceived social support	−0.265**	1	
Sense of agency	−0.275**	0.362**	1
Number of WMSD sites	0.387**	−0.308**	−0.337**

** *p* < 0.001.

### 3.4 Construction and test of structural equation model of nurses’ WMSDs

Six control variables were included, including gender, length of service, night shift, frequent trunk flexion, frequent heavy lifting, and perceived work fatigue. The final model was obtained after parameter definition and parameter test. The model’s χ2 = 2934.26, df = 393, χ2/df = 7.466 (*p* < 0.001), CFI = 0.906, RMSEA = 0.070 (0.067–0.072), TLI = 0.780. While the TLI value fell below the conventional threshold of 0.90, the CFI and RMSEA values indicated an acceptable model fit. Given the complexity of the model (six control variables and three latent pathways) and the large sample size (*n* = 1,388), which tends to inflate χ^2^ values and decrement approximate fit indices, the CFI and RMSEA values supported by the significant and theoretically coherent path coefficients.

**Figure 1:**
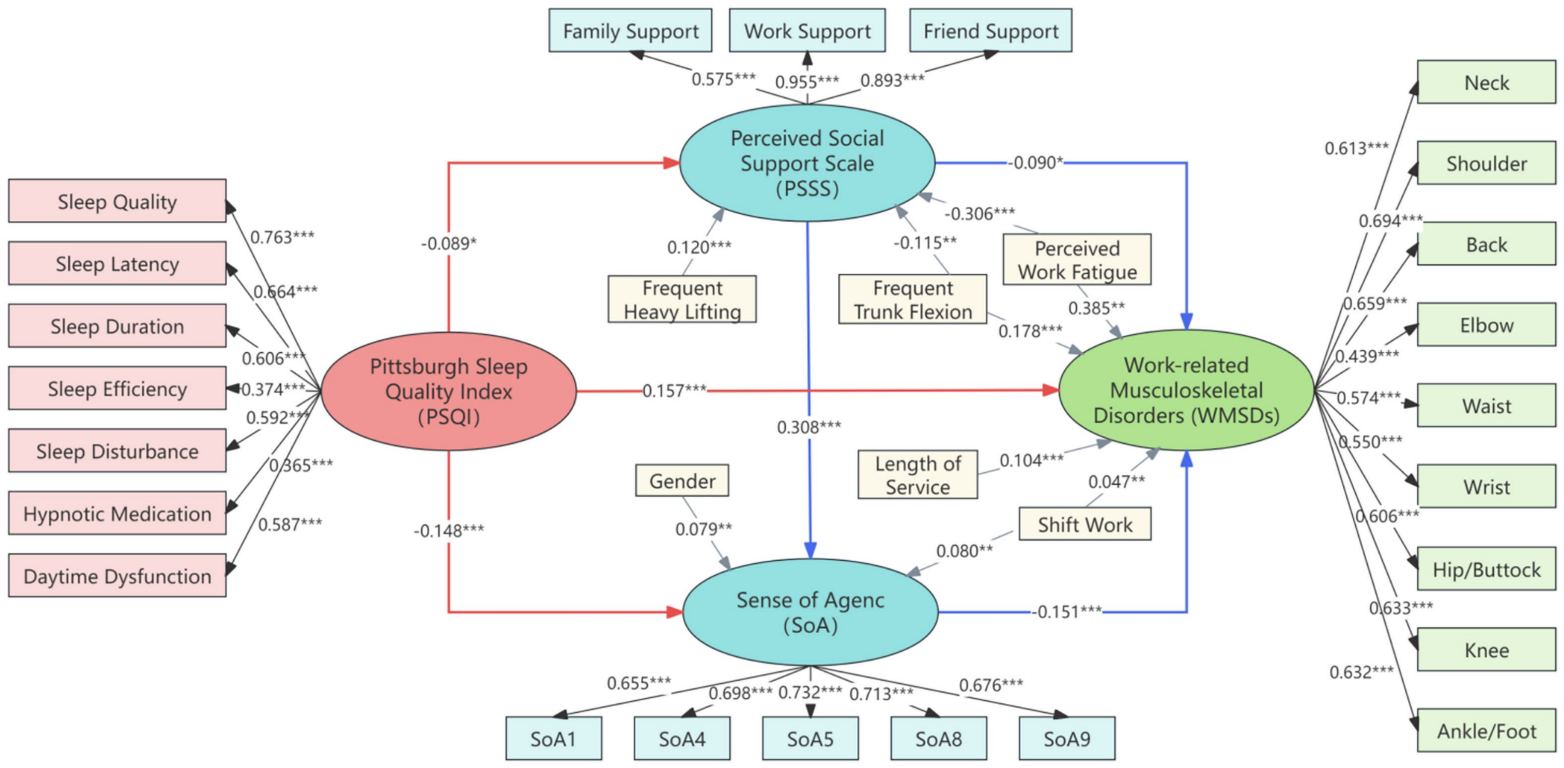
Standardized path coefficient diagram of sleep-social psychology-WMSDs of nurses. * *p* < 0.05, ** *p* < 0.01, *** *p* < 0.001.

The path coefficient of the model showed that the sleep quality index of nurses had a direct negative impact on the sense of agency and perceived social support (*p* < 0.05). The sleep quality index had a direct positive effect on nurses’ WMSDs (*p* < 0.05). Perceived support had a direct positive effect on sense of agency (*p* < 0.05). Perceived support and sense of agency had a direct negative effect on nurses’ WMSDs (*p* < 0.05). The specific situation is shown in Figure 1 and Table 3. The indirect effect of three significant paths accounted for the total effect ratio of 0.035/0.192 = 18.2%.

**Table 3:** Path coefficient of the effect of sleep quality on WMSDs in nurses.

Item			Estimate	S.E.	*P*	95%CI
The main path						
WMSDs	←	Sleep Quality Index	0.157	0.028	<0.001	(0.102, 0.213)
WMSDs	←	Perceived Social Support	−0.090	0.031	0.035	(−0.109, −0.015)
WMSDs	←	Sense of Agency	−0.151	0.034	<0.001	(−0.217, −0.085)
Sense of agency	←	Sleep Quality Index	−0.148	0.033	<0.001	(−0.214, −0.083)
Sense of agency	←	Perceived Social Support	0.308	0.041	<0.001	(0.228, 0.389)
Perceived social support	←	Sleep Quality Index	−0.089	0.03	0.003	(−0.146, −0.031)
The influence path of control variables						
WMSDs	←	Perceived work fatigue	0.385	0.03	<0.001	(0.327, 0.443)
WMSDs	←	Frequent trunk flexion	0.178	0.025	<0.001	(0.129, 0.228)
WMSDs	←	Length of service	0.104	0.027	<0.001	(0.052, 0.156)
WMSDs	←	Night shift	0.047	0.023	0.041	(0.002, 0.092)
Sense of agency	←	Gender	0.079	0.029	0.007	(0.021, 0.137)
Sense of agency	←	Night shift	0.08	0.027	0.003	(0.027, 0.132)
Perceived social support	←	Perceived work fatigue	−0.306	0.03	<0.001	(−0.364, −0.248)
Perceived social support	←	Frequent trunk flexion	−0.115	0.027	<0.001	(−0.167, −0.063)
Perceived social support	←	Frequent heavy lifting	−0.12	0.028	<0.001	(−0.174, −0.066)

## 4 Discussion

In the present study we observed that the poorer the sleep quality, the greater the number of anatomical sites affected by WMSDs reported by nurses, confirming Hypothesis 1. This finding aligns with recent evidence on the nexus between sleep and occupational health. First, the association between sleep quality on WMSDs is plausibly mediated by neuro-endocrine and physiological dysregulation consequent to sleep loss or fragmentation. Adequate sleep is indispensable for stabilizing neuromuscular function, facilitating tissue repair and modulating inflammation (22). Experimental work demonstrates that sleep deprivation or disrupted sleep architecture produces hyper-activation of the hypothalamic–pituitary–adrenal (HPA) axis, elevates cortisol secretion and propagates systemic inflammatory responses (23). Chronic low-grade inflammation not only lowers pain threshold and heightens sensitivity of muscular and articular tissues (24), but also impairs collagen metabolism and reparative capacity. Moreover, reduced slow-wave sleep (SWS) attenuates the nocturnal growth-hormone surge, ultimately manifesting as decreased muscle strength and persistence of WMSD symptoms (25). Among clinical nurses—who routinely perform night shifts, rotating schedules and high-intensity patient-handling tasks—the coexistence of sleep disorders further compromises recovery capacity and accelerates WMSD onset and progression. Second, poor sleep may indirectly magnify the physical and psychological burdens of the work environment by eroding affective regulation and coping resources (26). Prolonged sleep restriction disturbs autonomic balance, characterized by heightened sympathetic and diminished parasympathetic tone. This chronic physiological stress delays post-exertion heart-rate recovery and prolongs muscle-relaxation time after fatigue (27), thereby increasing local musculoskeletal loading. The direct association documented here remained robust after adjustment for years in nursing, shift-work status and physical workload, indicating that sleep quality is independently associated with WMSDs rather than a mere epiphenomenon. Improving sleep should therefore be positioned not simply as a component of quality-of-life or mental-health initiatives, but as a core element of evidence-based strategies for the primary prevention of occupational musculoskeletal disorders.

We further demonstrated that sense of agency mediates the relationship between sleep quality and WMSDs among nurses, corroborating Hypotheses 2 and 3. Sense of agency, defined as the subjective experience of initiating and regulating one’s own behavior and, through it, influencing external events, has been repeatedly linked to psychological well-being and stress regulation. In the present sample, sleep quality showed a significant association with nurses’ level of agency. The likely mechanism is that chronic sleep restriction or fragmentation compromises pre-frontal cortical function, erodes executive control and decisional capacity, and simultaneously intensifies emotional exhaustion and fatigue, all of which were associated with lower perceived autonomy and self-efficacy at work (28, 29). When perceived agency declines, nurses confronted with high-intensity, high-demand care tasks tend to display fewer adaptive coping behaviors; the precision and adaptability of movement execution decrease (30), which may expose the musculoskeletal system to sustained and biomechanically inappropriate loads and may be associated with higher WMSD risk. Theoretically, this mediated pathway integrates Conservation of Resources (COR) theory with the Job Demand–Control (JD-C) model. Sleep constitutes a fundamental physiological resource; its depletion drains the individual’s resource pool, including the cognitive resource of perceived agency. High job demands coupled with low perceived control (i.e., narrow decision latitude) trigger both psychological and physiological stress responses (31). By extending the JD-C framework into the domain of sleep health, our data confirm that sleep quality is a critical antecedent of the cognitive appraisal of control (32). Diminished agency is correlated with greater perceived pressure of job demands but may co-occur with sustained psychophysiological stress reactions, increased muscular tension and heightened pain sensitivity, that jointly constitute a psycho-physiological channel associated with WMSD development.

Concurrently, perceived social support emerged as a mediator between sleep quality and WMSDs, confirming Hypotheses 4 and 5. Nursing is inherently team-based and emotionally demanding; social support functions not merely as psychological comfort but as a structural resource that sustains occupational competence and physical safety. When sleep quality deteriorates, impaired affect regulation and heightened negative cognitive bias prevent nurses from adequately appraising available support, even when it objectively exists (33). Perceived support may function as a “buffering’ resource”: under high-strain conditions it attenuates stress reactivity and lowers cortisol secretion, thereby reducing chronic inflammation and muscular tension (34). Tangible assistance from colleagues and supervisors was associated with more equitable workload distribution and fewer reported WMSD sites (35). Perceived friend support mitigates social jet-lag and burnout, conferring additional protection against WMSDs (36, 37).

At the same time, our study observed that the direct effect of perceived social support on the number of WMSD sites (*β* = −0.090) was statistically significant, yet the effect size was small. First, compared with other variables in the model (e.g., perceived work fatigue, β = 0.385), the independent contribution of social support was indeed relatively limited, suggesting that social support may not function as a robust main-effect protective factor but rather operates more like a buffer or contextual resource. Second, a small effect in occupational epidemiology does not necessarily equate to being negligible in clinical or managerial significance. In the present model, social support not only exerted a direct association but, more importantly, served as a critical mediating node between sleep quality and sense of agency (*β* = 0.308, *p* < 0.001). However, within the “high fatigue + high mechanical exposure” subgroup the protective path was amplified, indicating that support operates chiefly when demands are elevated. The model also revealed a chained mediation through sense of agency, underscoring that weakened perceptions of organizational and team support erode nurses’ subjective beliefs and self-efficacy (38). Thus, when physiological and psychological resources are severely taxed, the social support network by delivering timely instrumental aid, credible information and emotional validation attenuates perceived stress, augmented coping efficacy, and fewer reported WMSD sites.

The influence of control variables on WMSDs also deserves attention. Sex differences in muscle strength, emotional reactivity and pain sensitivity render women more susceptible to musculoskeletal symptoms, a pattern consistent with the majority of previous reports (3). Moreover, sex exerted an indirect association with WMSDs via sense of agency, probably because women often shoulder simultaneous work and family roles, accelerating the depletion of psychological resources and predisposing them to perceived loss of control under high strain. Age and length of employment reflect cumulative occupational exposure, declining compensatory capacity and increased risk of chronic wear-and-tear lesions (39). Among work-related factors, shift duty not only predicted WMSDs directly, but also operated indirectly by eroding nurses’ sense of agency. Night and irregular rotating shifts disrupt endogenous circadian rhythms, provoke melatonin secretion disturbances, fragment sleep architecture and impair autonomic balance; the resulting accumulation of muscular fatigue and insufficient recovery directly amplifies musculoskeletal loading (40, 41). Simultaneously, the intrusion of shift work into personal life systematically undermines perceived mastery over work and life domains. When individuals repeatedly confront fatigued work states coupled with slowed decision-making, confidence in the effectiveness of their own actions gradually disintegrates (42). Frequent adoption of awkward postures constitutes the most immediate biomechanical factor associated with WMSDs, coinciding with over-use and symptoms in local muscles, ligaments, and joints (43). Perceived job fatigue, a subjective experience reflecting prolonged physical loading, manifests as energy depletion, diminished attention and impaired motor coordination, directly compromising trunk stability (44). Our data further indicate that increased fatigue perception weakened perceived social support. Individuals in a state of high fatigue possess limited cognitive and emotional resources, often displaying social withdrawal and emotional blunting; consequently they are less likely to seek support and less able to recognize and respond to supportive cues from colleagues (45).

In summary, while traditional WMSD research has focused primarily on ergonomic and biomechanical risk factors, potentially explaining limited variance in symptom outcomes when psychosocial variables are omitted, the integrated model constructed in this study advances the literature at two levels in terms of theoretical mechanism and explanatory depth. First, the framework integration. Whereas previous studies have predominantly examined unidirectional associations such as “sleep–WMSDs” or “support–WMSDs” in isolation, the present study demonstrates that sleep quality can simultaneously exert both direct and indirect (via support and sense of agency) associations with WMSDs, providing quantitative evidence for the application of the biopsychosocial medical model in the occupational health domain. Second, the identification of a chained mediation pathway. Our model reveals that perceived social support and sense of agency are not parallel mediators; rather, a longitudinal transmission pathway exists from “support → agency” (*β* = 0.308). This finding refines the conventional Job Demand–Control (JD-C) model regarding “job control/support”: social support not only directly buffers stress but also indirectly reduces WMSD risk by reshaping nurses’ perceived mastery over their own capabilities.

Nevertheless, several limitations should be acknowledged. First, the cross-sectional design precludes causal inference; although it allows us to delineate associations among sleep quality, perceived social support, sense of agency and WMSDs, it cannot establish temporal precedence. Second, all data were obtained by self-report questionnaires, which are vulnerable to recall and social-desirability biases. Additionally, the NMQ scoring used in this study captured the number of symptomatic regions rather than pain intensity, frequency, or functional impairment. Future investigations should incorporate objective sleep-monitoring indices and inflammatory biomarkers to strengthen the physiological underpinning of the proposed pathways.

## 5 Conclusion

Our findings demonstrate that sleep quality exerts both a direct positive effect on WMSDs and an indirect effect via psychosocial pathways. The results validate the applicability of a “physiological–psychological–occupational health” multiple-mediation model in the occupational-health arena and underscore that the development of WMSDs in nurses is not a purely biomechanical process, but rather a complex outcome of interactions between physiological and psychosocial factors. Future practice should adopt integrated health-promotion strategies: while optimizing shift schedules and improving rest facilities to protect sleep quality, equal attention must be paid to psychosocial interventions, fostering an organizational climate of support and increasing decision-making participation, to block the translation of sleep problems into musculoskeletal morbidity through multiple routes. Moreover, the proposed model can facilitate precise identification of high-risk individuals. A risk-stratification algorithm for nurse WMSDs can be constructed by weighting variables with the strongest association identified herein (perceived fatigue, shift work, years in nursing). Nurses who accumulate several of these factors can then be prioritized for targeted interventions.

## Data Availability

The raw data supporting the conclusions of this article will be made available by the authors, without undue reservation.

## References

[1] Amini Rarani S. Nursing workforce in collapse: a narrative review of global shortages, burnout, and the future of health system resilience. Int J Afr Nurs Sci. (2026) 24:100938. doi: 10.1016/j.ijans.2025.100938

[2] Aiken LH Lasater KB Sloane DM Pogue CA Fitzpatrick Rosenbaum KE Muir KJ . Physician and nurse well-being and preferred interventions to address burnout in hospital practice: factors associated with turnover, outcomes, and patient safety. JAMA Health Forum. (2023) 4:e231809. doi: 10.1001/jamahealthforum.2023.1809, 37418269 PMC10329209

[3] Wang K Zeng X Li J Guo Y Wang Z. The prevalence and risk factors of work-related musculoskeletal disorders among nurses in China: a systematic review and meta-analysis. Int J Nurs Stud. (2024) 157:104826–6. doi: 10.1016/j.ijnurstu.2024.104826, 38843644

[4] Santos W Lorente A Rojas C Isidoro R Dias A Mariscal G . A systematic review and meta-analysis on the prevalence and demographic risk factors of work-related musculoskeletal disorders in construction workers. Front Public Health. (2025) 13:1651921–1. doi: 10.3389/fpubh.2025.1651921, 41158564 PMC12554755

[5] Mahajan D Gupta MK Mantri N Joshi NK Gnanasekar S Goel AD . Musculoskeletal disorders among doctors and nursing officers: an occupational hazard of overstrained healthcare delivery system in western Rajasthan, India. BMC Musculoskelet Disord. (2023) 24:349–9. doi: 10.1186/s12891-023-06457-z, 37142985 PMC10157123

[6] Amatori S Gobbi E Sisti D Pivato G Giombini G Rombaldoni R . Physical activity, musculoskeletal disorders, burnout, and work engagement: a cross-sectional study on Italian white-collar employees. Front Public Health. (2024) 12:1375817–7. doi: 10.3389/fpubh.2024.1375817, 38746006 PMC11091297

[7] Zhao X Zhang L Zhang X Guo J Guan R Cheng Y . The prevalence and risk factors of shift work disorder among nurses: a systematic review and meta-analysis. Int J Nurs Stud. (2025) 174:105273. doi: 10.1016/j.ijnurstu.2025.105273, 41223687

[8] Niu J An Y Xu M Zhang L Liu J Feng X . Do sleep and psychological factors influence musculoskeletal pain among nurses? Work. (2023) 75:1455–65. doi: 10.3233/WOR-211113, 36710694

[9] Hertel E Sathiyalingam E Pilgaard L Brommann SJ Giordano R Petersen KKS. Psychophysical changes after total sleep deprivation and experimental muscle pain. J Sleep Res. (2025) 34:e14329–9. doi: 10.1111/jsr.14329, 39289848 PMC11911060

[10] Xie W Liu M Okoli CTC Zeng L Huang S Ye X . Construction and evaluation of a predictive model for compassion fatigue among emergency department nurses: a cross-sectional study. Int J Nurs Stud. (2023) 148:104613. doi: 10.1016/j.ijnurstu.2023.104613, 37839306

[11] Bezzina A Austin E Nguyen H James C. Workplace psychosocial factors and their association with musculoskeletal disorders: a systematic review of longitudinal studies. Workplace Health Saf. (2023) 71:578–88. doi: 10.1177/21650799231193578, 37698343 PMC10676046

[12] Hurault J Hurault J-C Broc G Crône L Tedesco A Brunel L. Measuring the sense of agency: a French adaptation and validation of the sense of agency scale (F-SoAS). Front Psychol. (2020) 11:584145–5. doi: 10.3389/fpsyg.2020.584145, 33132992 PMC7579422

[13] Zimmerman ME Benasi G Hale C Yeung LK Cochran J Brickman AM . The effects of insufficient sleep and adequate sleep on cognitive function in healthy adults. Sleep Health. (2024) 10:229–36. doi: 10.1016/j.sleh.2023.11.011, 38233280 PMC11045317

[14] Scott AJ Webb TL Martyn-St James M Rowse G Weich S. Improving sleep quality leads to better mental health: a meta-analysis of randomised controlled trials. Sleep Med Rev. (2021) 60:101556–6. doi: 10.1016/j.smrv.2021.101556, 34607184 PMC8651630

[15] Cao Y Xie T Ma N. The impairments of sleep loss on core executive functions: general and task-specific effects. Sleep Med Rev. (2025) 84:102163–3. doi: 10.1016/j.smrv.2025.102163, 40946426

[16] Piras I Usai V Contu P Galletta M. Vicarious trauma, coping strategies and nurses' health outcomes: An exploratory study. AIMS Public Health. (2024) 11:1071–81. doi: 10.3934/publichealth.2024055, 39802564 PMC11717540

[17] AbdELhay ES Taha SM el-Sayed MM Helaly SH AbdELhay IS. Nurses retention: the impact of transformational leadership, career growth, work well-being, and work-life balance. BMC Nurs. (2025) 24:148–8. doi: 10.1186/s12912-025-02762-1, 39923025 PMC11807322

[18] Ren J Zhang X Gao Y. Influencing factors of work engagement among ophthalmic specialized nurses in China: a cross-sectional study. BMC Nurs. (2024) 23:795–5. doi: 10.1186/s12912-024-02452-4, 39478542 PMC11523766

[19] Asuquo EG Murphy-Tighe S Ryan R O'Sullivan K. How is social support defined, categorized and measured in studies of work-related musculoskeletal disorders among hospital nurses: a scoping review. J Adv Nurs. (2025) 81:1130–41. doi: 10.1111/jan.16356, 39164033 PMC11810495

[20] Geto AK Daba C Desye B Berihun G Berhanu L. Prevalence of work-related musculoskeletal disorder and its associated factors among weavers in low- and middle-income countries: a systematic review and meta-analysis. BMJ Open. (2025) 15:e093124–4. doi: 10.1136/bmjopen-2024-093124, 40754326 PMC12320042

[21] Tapal A Oren E Dar R Eitam B. The sense of agency scale: a measure of consciously perceived control over one's mind, body, and the immediate environment. Front Psychol. (2017) 8:1552–2. doi: 10.3389/fpsyg.2017.01552, 28955273 PMC5600914

[22] Angelini CI Siciliano G Ansevin C. Editorial: sleep disorders in neuromuscular diseases: treatable conditions: the evolving scenario of sleep in neuromuscular disorders. Front Neurol. (2024) 15:1448486–6. doi: 10.3389/fneur.2024.1448486, 39055315 PMC11270873

[23] Stenger S Vorobyev A Bieber K Lange T Ludwig RJ Hundt JE. Insomnia increases the risk for specific autoimmune diseases: a large-scale retrospective cohort study. Front Netw Physiol. (2025) 5:1499297–7. doi: 10.3389/fnetp.2025.1499297, 40276126 PMC12018472

[24] Yin Y Zhang X. The causal relationship between sleep characteristics and multi-site pain perception: a two-sample mendelian randomization study. Front Neurosci. (2024) 18:1428951–1. doi: 10.3389/fnins.2024.1428951, 39193526 PMC11347297

[25] Easow J Bommasamudram T Munnilari M Adhikari R Edwards BJ Nayak KR . Implications of sleep loss or sleep deprivation on muscle strength: a systematic review. Sleep Breath. (2025) 29:242–2. doi: 10.1007/s11325-025-03413-0, 40663194 PMC12263768

[26] Khcharem A Masmoudi L Sahnoun Z Sahli S. Sleep loss costs performance: physical, cognitive, and psychological impairments after 26 h of sleep deprivation in student athletes. Chronobiol Int. (2026) 43:377–386. doi: 10.1080/07420528.2025.2600581 41363293

[27] Saputro RE Chou CC Lin YY Tarumi T Liao YH. Exercise-mediated modulation of autonomic nervous system and inflammatory response in sleep-deprived individuals: a narrative reviews of implications for cardiovascular health. Auton Neurosci. (2025) 259:103256. doi: 10.1016/j.autneu.2025.103256, 40073691

[28] Zhao K Dang J Gu J Fu X Haggard P. Cognitive mechanisms underlying sense of agency: Meta-analytic reviews of behavioral and neuroimaging studies. Psychol Bull. (2025) 151:1307–35. doi: 10.1037/bul0000497, 41428510

[29] Zhang K Li P Zhao Y Griffiths MD Wang J Zhang MX. Effect of social media addiction on executive functioning among young adults: the mediating roles of emotional disturbance and sleep quality. Psychol Res Behav Manag. (2023) 16:1911–20. doi: 10.2147/PRBM.S414625, 37255996 PMC10226546

[30] Puech L Moutsopoulou K Brunel L. Sense of agency and ideomotor learning: high dispositional sense of agency is linked to better action-effect learning. Q J Exp Psychol. (2006) 2025:17470218251365225–5. doi: 10.1177/17470218251365225 40751571

[31] Voss AS Soucek R Moser K Drexler H. The differentiated roles of resilient behavior and job crafting in interaction with work intensity and their impact on employee health and performance. Int J Environ Res Public Health. (2025) 22:429. doi: 10.3390/ijerph22030429, 40238562 PMC11942536

[32] Opoku MA Kang S Choi SB. The influence of sleep on job satisfaction: examining a serial mediation model of psychological capital and burnout. Front Public Health. (2023) 11:1149367–7. doi: 10.3389/fpubh.2023.1149367, 37693724 PMC10483141

[33] Baker C Kirby JB O'Connor J Lindsay KG Hutchins A Harris M. The perceived impact of ashwagandha on stress, sleep quality, energy, and mental clarity for college students: qualitative analysis of a double-blind randomized control trial. J Med Food. (2022) 25:1095–101. doi: 10.1089/jmf.2022.0042, 35984870

[34] Aw TBH Zainal NH. Interleukin-6 moderates the relationship between social support, strain, and future depressive symptoms. Brain Behav Immun Health. (2025) 49:101122–2. doi: 10.1016/j.bbih.2025.101122, 41210298 PMC12590439

[35] Albanesi B Piredda M Bravi M Bressi F Gualandi R Marchetti A . Interventions to prevent and reduce work-related musculoskeletal injuries and pain among healthcare professionals. A comprehensive systematic review of the literature. J Saf Res. (2022) 82:124–43. doi: 10.1016/j.jsr.2022.05.004, 36031239

[36] Pergol-Metko P Staniszewska A Metko S Sienkiewicz Z Czyzewski L. Compassion fatigue and perceived social support among polish nurses. Healthcare (Basel). (2023) 11:706. doi: 10.3390/healthcare11050706, 36900712 PMC10001227

[37] Shen Y Zhao M Wei N Zhao W Han M Dai S . Associations among social jet lag, sleep-related characteristics, and burnout of nurses in tertiary hospitals. Holist Nurs Pract. (2024) 38:385–93. doi: 10.1097/HNP.0000000000000637, 38451845

[38] Kim E Lee G Lee JE Martin P. Reciprocal relationship between sense of control and social support: a random-intercept cross-lagged panel model. Aging Ment Health. (2025) 29:1525–34. doi: 10.1080/13607863.2025.2484354, 40159908 PMC12213213

[39] Yin L Fan L Liang J Tan J Yan Y Huang L . Prediction model and assessment of work-related musculoskeletal disorders among nurses in tertiary hospitals in China: a cross-sectional study. Br J Hosp Med. (2005) 86:1–24. doi: 10.12968/hmed.2024.1045 40847979

[40] Rosa DE Marot LP de Mello MT Narciso FV Gonçalves BSB Marqueze EC . Shift rotation, circadian misalignment and excessive body weight influence psychomotor performance: a prospective and observational study under real life conditions. Sci Rep. (2019) 9:19333–3. doi: 10.1038/s41598-019-55114-w, 31852906 PMC6920148

[41] Panwar A Bagla RK Mohan M Rathore BB. Influence of shift work on sleep quality and circadian patterns of heart rate variability among nurses. J Family Med Prim Care. (2024) 13:3345–9. doi: 10.4103/jfmpc.jfmpc_158_24, 39228548 PMC11368331

[42] Lee A Lin YK Lin YH Chang WP. A longitudinal study of rotating shift type and attention performance of acute and critical care nurses with chronotype as moderator variable. J Occup Health. (2024) 66:uiae042. doi: 10.1093/joccuh/uiae042, 39038080 PMC11360591

[43] Liu F Duan Y Wang Z Ling R Xu Q Sun J . Mixed adverse ergonomic factors exposure in relation to work-related musculoskeletal disorders: a multicenter cross-sectional study of Chinese medical personnel. Sci Rep. (2025) 15:14705–5. doi: 10.1038/s41598-025-99477-9, 40289235 PMC12034785

[44] Magnuson JR Doesburg SM McNeil CJ. Development and recovery time of mental fatigue and its impact on motor function. Biol Psychol. (2021) 161:108076–6. doi: 10.1016/j.biopsycho.2021.108076, 33716108

[45] Liu L Wu D Wang L Qu Y Wu H. Effort-reward imbalance, resilience and perceived organizational support: a moderated mediation model of fatigue in Chinese nurses. Risk Manag Healthc Policy. (2020) 13:893–901. doi: 10.2147/RMHP.S259339, 32801964 PMC7394598

